# Modulation of Arm Reaching Movements during Processing of Arm/Hand-Related Action Verbs with and without Emotional Connotation

**DOI:** 10.1371/journal.pone.0104349

**Published:** 2014-08-05

**Authors:** Silvia Spadacenta, Vittorio Gallese, Michele Fragola, Giovanni Mirabella

**Affiliations:** 1 PhD Program in Neurophysiology, Department of Physiology and Pharmacology, University of Rome La Sapienza, Rome, Italy; 2 Department of Neuroscience, University of Parma, Parma, Italy; 3 Department of Physiology and Pharmacology, University of Rome La Sapienza, Rome, Italy; 4 Istituto Neurologico Mediterraneo Neuromed, Pozzilli (IS), Italy; Birkbeck College, United Kingdom

## Abstract

The theory of embodied language states that language comprehension relies on an internal reenactment of the sensorimotor experience associated with the processed word or sentence. Most evidence in support of this hypothesis had been collected using linguistic material without any emotional connotation. For instance, it had been shown that processing of arm-related verbs, but not of those leg-related verbs, affects the planning and execution of reaching movements; however, at present it is unknown whether this effect is further modulated by verbs evoking an emotional experience. Showing such a modulation might shed light on a very debated issue, i.e. the way in which the emotional meaning of a word is processed. To this end, we assessed whether processing arm/hand-related verbs describing actions with negative connotations (e.g. to stab) affects reaching movements differently from arm/hand-related verbs describing actions with neutral connotation (e.g. to comb). We exploited a go/no-go paradigm in which healthy participants were required to perform arm-reaching movements toward a target when verbs expressing emotional hand actions, neutral hand actions or foot actions were shown, and to refrain from moving when no-effector-related verbs were presented. Reaction times and percentages of errors increased when the verb involved the same effector as used to give the response. However, we also found that the size of this interference decreased when the arm/hand-related verbs had a negative emotional connotation. Crucially, we show that such modulation only occurred when the verb semantics had to be retrieved. These results suggest that the comprehension of negatively valenced verbs might require the simultaneous reenactment of the neural circuitry associated with the processing of the emotion evoked by their meaning and of the neural circuitry associated with their motor features.

## Introduction

The way in which concepts are represented is still much debated. On the one hand classical theories of cognition [1] or the more recent so-called ‘disembodied cognition hypothesis’ (see [2]) state that conceptual representations are amodal or symbolic (linguistic), i.e. they are qualitatively distinct and entirely separated from sensory and motor information. On the other hand the embodied theory of cognition asserts that concepts are grounded in bodily experience and thus their understanding relies on an internal reenactment of the sensorimotor experience associated with that concept (e.g. [3]–[5]). Therefore, according to the disembodied cognition hypothesis the comprehension of the concept expressed by the verb ‘to cut’ would rely on the retrieval of a symbolic representation released from any link with sensorimotor experience. In contrast, the theory of embodied cognition asserts that the understanding of the verb ‘to cut’ would be obtained by an internal reenactment of the sensorimotor experience associated with the action described by the verb (with or without the concomitant activation of other brain areas; see [6] for a review).

Recently it has been shown that the processing of arm/hand-related verbs specifically interferes with arm-reaching movements in a go/no-go task only when word semantics have to be retrieved [7]. In the experiments, participants were asked to perform two versions of a go/no-go task. In one version of the task, participants had to perform arm-reaching movements toward a visual target when verbs expressing either hand or foot actions were shown, and to refrain from moving when no-effector-related verbs were presented (semantics task). It was found that when the verbs described an action involving the same effector as used to give the response, i.e. the arm, RTs were lengthened and the number of errors significantly increased with respect to when the verbs described leg-related actions (interference effect). In the other version of the task, the same verbs were presented but this time participants had to decide whether to move or to stop according to the color in which the verbs were printed, disregarding their meaning. Under these conditions any difference between arm/hand- and leg/foot-related verbs disappeared. Even though it could be not assessed directly, these results seem to suggest that a somatotopic recruitment of effector-related representations in the motor system occurs only when the word's semantics has to be retrieved, and thus are fully compatible with the embodied theory of language (see also [8]).

It is noteworthy that all verbs employed in the described study referred to concrete actions with a clear sensorimotor grounding. Thus, as in many other studies in this field (see [4] for a review), it remained undetermined whether the representation of more abstract concepts could also rely on embodied mechanisms. Relevant to this issue are the findings of Kousta et al. ([9], recently replicated by [10]). They reported that, when other confounding variables were controlled, lexical decision latencies were faster for abstract words than for concrete words, in contrast to the more common result whereby reaction times (RTs) are shorter for concrete than for abstract words. Crucially, Kousta et al. [9] demonstrated that the processing advantage of abstract words was due to their higher hedonic valence with respect to that of concrete items. Analogous results were obtained by Newcombe et al. [11] exploiting a semantic categorization task in which participants had to perform a key-press either to the presentation of abstract nouns or of concrete nouns according to the instruction of the experimenters. These findings are consistent with Vigliocco et al.'s [4] embodied theory of semantic representation, according to which sensorimotor experience underlines the processing of concrete concepts whereas emotional experience underlines the processing of abstract concepts. In other words, while sensorimotor experience would be crucial for the embodiment of concrete concepts, affective experience would play a key role in the embodiment of abstract concepts. According to this view, the understanding of abstract concepts such as ‘beauty’ or ‘justice’ will be derived primarily from the reenactment of the associated emotional experience.

Some other studies have explored the relationship between the processing of concrete words with an emotional connotation and motor responses (muscle activation or action execution). The aim was to demonstrate that at least some words with an affective meaning are body-grounded. For instance, Havas et al. [14] showed that healthy participants more quickly understood pleasant sentences while holding a pen between the teeth (to induce smiling) than while holding a pen between the lips (to inhibit smiling), and *vice versa*. Thus, this study showed that the physical simulation of emotions facilitated or inhibited the processing of words with emotional valence, according to the congruence or incongruence of the action performed. In addition, Foroni & Semin [12] demonstrated that verbs referring to emotional expressions, such as smiling and frowning, elicit the same facial muscle activity that occurs when individuals express that emotion. Therefore they conclude that comprehending the meaning of concrete verbs describing facial expressions induces a motor resonance in the reader. Most importantly they also showed that motor resonance is used in the understanding of emotional experience because when it was prevented (e.g. when participants held a pen with their lips) it causally influenced the shaping of affective judgments. Finally, Chen & Bargh [13] have shown that the emotional valence of a word interferes with the execution of a movement. In fact, they found that participants were faster to identify positive words (e.g. love) when the response consisted of an approaching action (arm flexion) than when the response consisted of a distancing action (arm extension). The opposite held true for negative words (e.g. hate).

Despite these results, the way in which the emotional meaning of a word is understood is still very controversial. To address this issue, we studied how the processing of verbs describing concrete actions with negative and neutral valence influences the actual execution of a goal-directed movement. We employed negative verbs because it has been shown that humans preferentially attend to negative stimuli rather than positive or neutral ones (e.g. see [15]–[16]). This is probably because the ability to detect information with negative emotional value and to respond with an appropriate behaviour is critical for our survival, as it could prevent potential harm or unpleasant social interactions.

Thus, using the same go/no-go design as Mirabella et al. [7] we wanted to assess: 1) whether the interference effect is modulated differently when participants have to understand arm/hand-related verbs describing actions with negative connotation (e.g. to stab) with respect to when they have to comprehend arm/hand-related verbs describing actions with neutral connotation (e.g. to comb); 2) whether such a modulation occurs only when verb semantics must be retrieved. It would greatly favor the embodied perspective if it were shown that, during language processing, the need to retrieve the emotional meaning of action verbs shapes motor actions in a way that differs from the situation with emotionally neutral verbs.

## Materials and Methods

### 1.1. Ethics statement

The experimental procedures were approved by the ethics board of the IRCCS Neuromed hospital and performed in accordance with the ethical standards laid down in the 1964 Declaration of Helsinki. All participants gave written informed consent. The following data will be freely available to anyone who will request them from the ethics committee of the IRCCS Neuromed hospital. As this study involve humans, public availability would compromise individual privacy.

### 1.2. Subjects

Thirty participants participated in Experiments 1 and 2 for course credits (mean age ± SEM: 25.2±0.6 years). All participants were native Italian speakers and were right-handed as assessed with the Edinburgh handedness inventory [17]. They had normal or corrected-to-normal vision and no history of speaking disorders. None of them was informed about the purpose of the experiments.

### 1.3. Verbal Stimuli

For Experiment 1, we selected twenty-four Italian verbs in the infinitive form (see Table 1), because in this form verbs engage only lexical–semantic retrieval processes whereas the inflected forms also engage syntactic and morphophonological integration processes (see [18]). Twelve verbs were arm/hand-related verbs: six described arm/hand-related actions with neutral connotations (e.g. “pettinare”: to comb) and six referred to hand/arm-related actions with negative emotional connotations (e.g. “accoltellare”: to stab). Six verbs described leg/foot-related actions (e.g. “marciare”: to march). Finally six verbs had a meaning which did not clearly involve movement of the effectors (e.g. “evaporare”: to evaporate). It has to be remarked that in the Italian language verbs describing leg/foot-related actions with a negative emotional valence as high as those describing arm/hand related action do not exist Therefore, unfortunately, it was not possible to employ them for a control experiment.

**Table 1:** Properties of stimuli used in the semantic task.

	Verbs	Letters	Syllable	Lexical Frequency	Imageability	Arousal	Valence	A/H vs L/F Relatedness
**Emotional arm/hand-related verbs**	Fucilare [to shoot]	8	4	7	6.50	4.47	−6.95	6.6
	Accoltellare [to stab]	12	5	11	6.04	5.20	−6.8	6.77
	Frustare [to whip]	8	3	6	6.23	4.27	−6.5	6.65
	Strozzare [to throttle]	9	3	14	6.15	5.40	−6.65	6.42
	Strangolare [to strangle]	11	4	22	6.19	5.20	−6.8	6.67
	Sgozzare [to slit one's throat]	8	3	11	6.12	4.60	−6.85	6.52
	**MEAN (±SEM)**	**9.33±0.7**	**3.66±0.3**	**11.8±2.36**	**6.2±0.06**	**4.85±0.2**	**6.75±0.1**	**6.6±0.05**
**Neutral arm/hand-related verbs**	Temperare [to sharpen]	9	4	6	6.50	0.87	−0.15	5.67
	Pettinare [to comb]	9	4	11	6.85	2.33	0	6.64
	Impastare [to knead]	9	4	16	6.73	1.73	0	6.4
	Strofinare [to scrub]	10	4	26	6.81	2.00	−0.6	5.55
	Massaggiare [to massage]	11	4	16	6.88	5.00	−0.02	5.95
	Colorare [to color]	8	4	29	6.96	3.07	0	6
	**MEAN (±SEM)**	**9.33±0.4**	**4±0.01**	**17.33±3.6**	**6.79±0.07**	**2.5±0.6**	**0.13±0.1**	**6.04±0.2**
**Leg/foot -related verbs**	Zoppicare [to limp]	9	4	10	6.69	3.20	−2.35	−6.6
	Marciare [to march]	8	3	45	6.62	1.80	−1.15	−6.22
	Pattinare [to skate]	9	4	4	6.85	3.20	0	−6.57
	Saltellare [to hop]	10	4	6	6.65	2.67	−0.2	−6.5
	Scavalcare [to leap over]	10	4	42	6.81	1.73	−0.97	−4.6
	Pedalare [to cycle]	8	4	37	6.96	2.47	−0.12	−6.57
	**MEAN (±SEM)**	**9.00±0.4**	**3.83±0.2**	**24±7.9**	**6.76±.0.05**	**2.51±.0.26**	**−0.8±0.4**	**−6.18±0.3**
**No-effector-related verbs**	Evaporare [to evaporate]	9	5	21	4.58	0.87	−0.35	0
	Memorizzare [to memorize]	11	5	12	4.58	3.27	−0.02	0
	Rinfrescare [to refresh]	11	4	6	5.77	3.13	0	0.67
	Persistere [to persist]	10	4	11	3.08	3.27	−0.72	0
	Sgorgare [to spring]	8	3	12	5.23	2.33	−0.2	0.4
	Ubbidire [to obey]	8	4	18	4.88	3.73	−0.85	0
	**MEAN (±SEM)**	**9.5±0.6**	**4.17±0.3**	**13.33±2.19**	**4.69±0.4**	**2.77±0.4**	**0.36±0.1**	**0.18±0.12**

For each item the number of letters, number of syllables, lexical frequency, imageability, arousal and English translation are given. Mean number of letters, syllables, lexical frequency, imageability, arousal, valence, and arm/hand vs. leg/foot (A/H vs L/F) relatedness (±SEM) are reported separately for each verb category.

Items were chosen from a list of 80 verbs (20 arm/hand-related verbs with negative connotation, 20 arm/hand-related and 20 leg/foot-related verbs with neutral connotation and finally 20 verbs with meaning which did not clearly involve movement of the effectors) on the basis of their unpleasantness–neutrality and on the basis of the effector used to perform the action. Forty participants, who did not take part in the experiment, completed a questionnaire in which the emotional valence of each selected verb was evaluated on an eight-point scale (−7 meant ‘very unpleasant’ and 0 neutral). The final list was composed of the eight verbs with the highest negative scores and 24 verbs with the scores closest to zero. A one-way analysis of variance (ANOVA; factor: verb categories) on valence revealed a main effect [F(3,20) = 240.02, p<0.0001]. Post hoc tests (pairwise comparisons with Bonferroni correction) showed that it was due to a significant difference between hand/arm-related verbs with negative emotional connotations and all the other verb categories (all p<0.0001). Verbs were matched for syllable number, word length and total lexical frequency (i.e. number of occurrences per ∼4,000,000 words; [19]). A one-way ANOVA (factor: verb categories) showed no significant differences between verb categories for syllable number [F(3,20) = 0.79) = , p = 0.51], word length [F(3,20) = 0.15, p = 0.93] or lexical frequency [F(3,20) = 1.39, p = 0.27].

In addition, 30 participants, who did not take part in the experiment, rated the imageability and the arousal of all verbs through questionnaires. Both imageability and arousal were rated on a scale ranging from 0 to 7. If the imageability of a verb was rated 0 it meant that it was “impossible to imagine”, while a value of 7 meant that the verb was “very easy to imagine”. We explained arousal to participants as follows: “If a word is arousing, it reflects a stimulated, excited, jittery or wide-awake state of feeling. If a word is not arousing, it reflects a relaxed, calm, sluggish or sleepy state of feeling” [20]. If the arousal of a verb was rated 0, it meant that it elicited a “state of feeling completely calm”, while a value of 7 meant that the verb elicited a “state of feeling stimulated”. A one-way ANOVA (factor: verb categories) showed a main effect of imageability [F(3,20) = 24.6, p<0.0001]. Post hoc tests (pairwise comparisons with Bonferroni correction) revealed that the imageabilities of arm/hand-related verbs (both emotional and neutral) and leg-related ones did not differ (emotional vs. neutral: p = 0.1; emotional vs. leg/foot: p = 0.46; neutral vs. leg/foot, p = 0.1), but they both had a different imageability from no-effector-related verbs (all p<0.0001). The same analysis performed on arousal showed a main effect [(F(3,20) = 8.35, p<0.001)]. *Post hoc* tests (pairwise comparisons with Bonferroni correction) showed, as expected, that arm/hand-related verbs with emotional meaning were significantly different from all other categories (all p<0.01).

Finally, in order to assess whether the verbs we used were clearly separable into arm/hand-related, foot/leg-related and no-effector-related verbs we administered a questionnaire to 50 subjects who did not take part in the experiment. On a scale ranging from −7 to 7, participants were required to score values i) bigger than zero when they considered the verb as describing an action performed with the arm/hand, ii) smaller than zero when they considered the verb as describing an action performed with the leg/foot and iii) around zero when they considered the verb as describing an action which did not clearly involve movement of the effectors (see Table 1). A one-way ANOVA (factor: verb categories) showed a main effect of effector relatedness [(F(3,20) = 1030.56, p<0.0001)]. Post hoc tests (pairwise comparisons with Bonferroni correction) showed, as expected, that arm/hand-related verbs with neutral and emotional meaning were not significantly different from each other (p = 0.19). However, they were both different from leg/foot- and no-effector-related verbs (all p<0.0001).

In Experiment 2, three verbs of each category (i.e. 12 verbs overall) were selected from the list used in Experiment 1 and were matched for syllable number, word length and lexical frequency. (see Table 2). One-way ANOVA (factor: verb categories) showed no significant differences between verb categories for syllable number [F(3,8) = 0.25, p = 0.86], word length [F(3,8) = 0.81, p = 0.52] or lexical frequency [F(3,8) = 0.17, p = 0.99]. In contrast, the one-way ANOVA (factor: verb categories) on imageability revealed a main effect [F(3,8) = 18.02, p<0.001]. *Post hoc* tests (pairwise comparisons with Bonferroni correction) showed that it was due to a significant difference between no-effector-related and action-related verbs (all p<0.01). Also, the one-way ANOVA (factor: verb categories) on arousal revealed a main effect [F(3,8) = 11.7, p<0.005]. As expected, this was due to a significant difference between arm/hand-related verbs with emotional meaning and the other categories (all p<0.05). We used only half of the verbs in each category to reduce the overall number of trials.

**Table 2:** Properties of stimuli used in color discrimination task.

	Verbs	Letters	Syllable	Lexical Frequency	Imageability	Arousal	Valence	A/H vs L/F Relatedness
**Emotional arm/hand-related verbs**	Accoltellare [to stab]	12	5	11	6.04	5.20	−6.8	6.77
	Strangolare [to strangle]	11	4	22	6.19	5.20	−6.8	6.67
	Sgozzare [to slit one's throat]	8	3	11	6.12	4.60	−6.85	6.52
	**MEAN (±SEM)**	**10.3±1.2**	**4±0.58**	**14.7±3.7**	**6.12±0.04**	**5±0.2**	**6.81±0.01**	**6.66±0.07**
**Neutral arm/hand-related verbs**	Temperare [to sharpen]	9	4	6	6.50	0.87	−0.15	5.67
	Pettinare [to comb]	9	4	11	6.85	2.33	0	6.64
	Colorare [to color]	8	4	29	6.96	3.07	0	6
	**MEAN (±SEM)**	**8.67±0.3**	**4**	**15.3±6.9**	**6.77±0.07**	**2.09±0.6**	**0.05±0.05**	**6.03±0.2**
**Leg/foot -related verbs**	Pattinare [to skate]	9	4	4	6.85	3.20	0	−6.57
	Saltellare [to hop]	10	4	6	6.65	2.67	−0.2	−6.5
	Pedalare [to cycle]	8	4	37	6.96	2.47	−0.12	−6.57
	**MEAN (±SEM)**	**9.00±0.6**	**4**	**15.7±10.7**	**6.82±.0.09**	**2.78±.0.2**	**−0.1±0.06**	**−6.55±0.03**
**No effector-related verbs**	Evaporare [to evaporate]	9	5	21	4.58	0.87	−0.35	0
	Memorizzare [to memorize]	11	5	12	4.58	3.27	−0.02	0
	Rinfrescare [to refresh]	11	4	6	5.77	3.13	0	0.67
	**MEAN (±SEM)**	**10.3±0.7**	**4.67±0.3**	**13±4.36**	**4.98±0.4**	**2.42±0.8**	**−0.13±0.1**	**0.22±0.2**

For each item the number of letters, number of syllables, lexical frequency, imageability, arousal and English translation are given. Mean number of letters, syllables, lexical frequency, imageability, arousal, valence, and arm/hand vs. leg/foot (A/H vs L/F) relatedness (±SEM) are reported separately for each verb category.

In Experiment 3, we used the 24 action-related verbs exploited in Experiment 1 (six neutral arm/hand-related; eight emotional arm/hand-related and eight leg/foot-related verbs) as real words. Pseudo-words were produced by replacing one consonant into the first or second syllable of each verb (i.e. “scucire” [to unsew], was changed into “scumire”). All the pseudo-words were pronounceable.

### 1.4. Behavioral tasks

Participants were required to perform two or three experiments. The order of administration was counterbalanced across participants. Tasks were completed in either one or two different experimental sessions occurring on different but consecutive days.

#### 1.4.1. Experiment 1 (semantic task)

**Figure 1:**
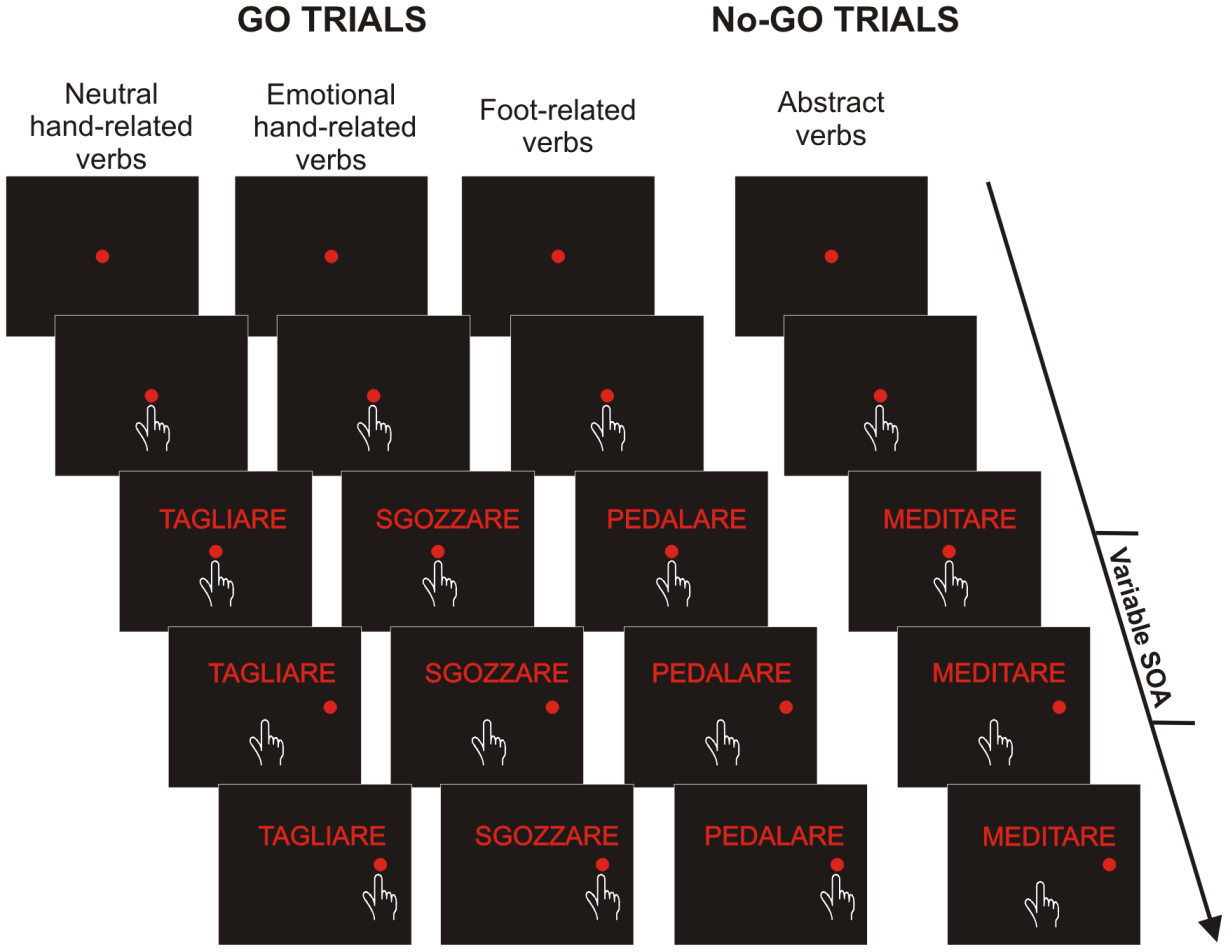
Schematic representation of semantic task. Each trial started with the presentation of a central red circle that participants had to touch and hold for a variable period. Then, a verb was shown above the central stimulus. After a variable delay (stimulus onset asynchrony; SOA) a peripheral target appeared. Participants were asked either to touch it, if the meaning of the verb referred to a concrete action (go-trials), or to refrain from moving if it had a content which did not clearly involve movement of the effectors (no-go trials; see Methods for more details).

The experiment was carried out in a sound-attenuated and dimly illuminated room. Participants sat comfortably at about 50 cm from a 17-inch PC monitor (CRT non-interlaced, refresh rate 75 Hz, 640×480 resolution, 32-bit color depth) equipped with a touch screen (MicroTouch; sampling rate 200 Hz) for touch-position monitoring. A noncommercial software package, CORTEX (http://www.brown.edu/Research/monkeylogic/), was used to control stimulus presentation and to collect behavioral responses. The temporal arrangements of stimulus presentation were synchronized with the monitor refresh rate. Participants performed the task with the right arm. Each trial began with the presentation of a central red circle (diameter: 3.2 degrees of visual angle, dva, or 2.8 cm) that participants had to touch with their index finger and to maintain touch) for a variable period (400–700 ms). Thereafter, a verb was presented just above the central stimulus and participants were instructed to carefully read it. When the verb referred to an action (go trials) participants had to reach and hold for a variable period (300–400 ms) a peripheral red circle (3.2 dva or 2.8 cm diameter) appearing on the right side of the screen at an eccentricity of 9.1 dva (or 8 cm). Conversely, when the verb described an action which did not clearly imply the use of an effector (no-go trials) participants had to keep the index finger still on the central stimulus for 400–800 ms (Fig. 1). Successful trials were signaled by acoustic feedback. The go-signal, given by the presentation of the peripheral target, was delivered either 53.2 ms after the presentation of the verb (stimulus onset asynchrony; SOA) or at an SOA of 332.5 ms. The purpose of using these two SOAs was to obtain data comparable with those of our previous work [7] enabling us to assess the timing of the occurrence of eventual effects linked to verb processing. Verbs remained visible until the end of the trial. All verbs were printed in red and were presented against a dark background with uniform luminance (<0.01 cd/m^2^). Each verb was presented ten times for each SOA. To discourage participants from slowing down responses we set an upper RT limit for go-trials: every time RTs were longer that 600 ms, go-trials were signaled as errors and aborted (overtime reaching-trials). Overtime reaching-trials were kept for the analyses, with the exception of those trials with RTs longer than 800 ms (less than 0.8% of the overall overtime reaching-trials). The experiment consisted of 480 trials, run in four blocks. Verb presentation was randomized and error trials were repeated until participants completed the block. Very importantly, we instructed participants to move when action verbs appeared and to stop at the presentation of no-effector-related verbs, but we never explicitly stated that action verbs were subdivided into different categories.

#### 1.4.2. Experiment 2 (color discrimination task)

**Figure 2:**
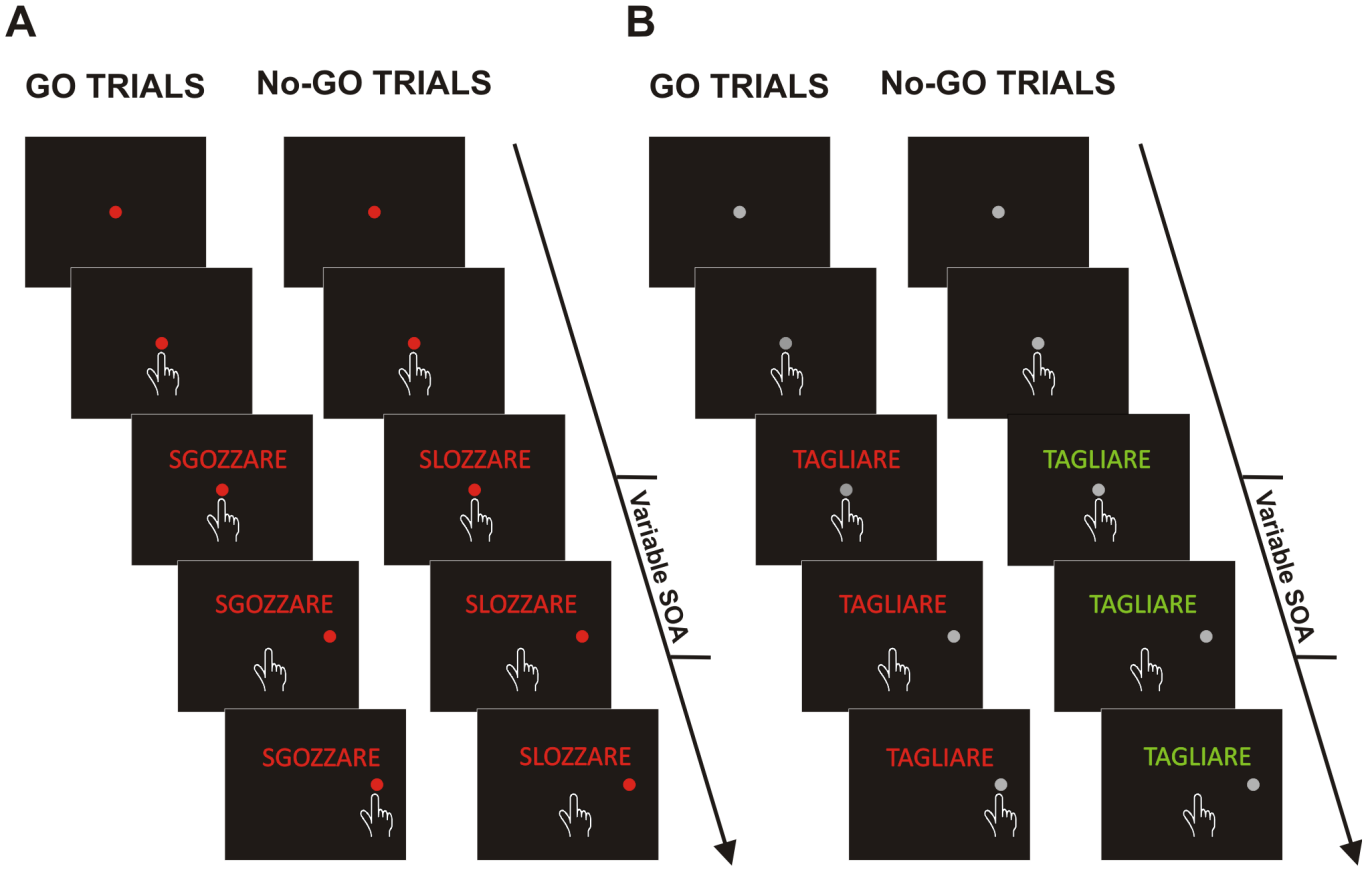
Schematic representation of control tasks. (A) Color discrimination task. Each trial started with the presentation of a red central target that participants had to touch and hold for a variable period. After a variable SOA a peripheral target appeared and participants were asked either to touch it if it was a real word (go-trials) or to stay still if it was a pseudo-word (no-go trials; see Methods for more details). **(B) Lexical task.** Each trial started with the presentation of a grey central target that participants had to touch and hold for a variable period. After a variable delay (stimulus onset asynchrony; SOA) a peripheral target appeared and participants were asked either to touch it, if it was printed in green (go-trials) or to stay still if it was printed in red (no-go trials; see Methods for more details).

In this experiment participants were instructed to execute or withhold arm movements according to the color in which verbs were printed. Each trial started with the presentation of a central stimulus (a grey circle with a diameter of 3.2 dva or 2.8 cm) that participants had to touch and hold for a variable period (400–700 ms). Thereafter, a verb was displayed above the central stimulus. When the verb was printed in green, participants were instructed to reach, as fast as possible, the peripheral target (a grey circle with a diameter of 3.2 dva or 2.8 cm) which was presented on the right side with an eccentricity of 9.1 dva (or 8 cm). Conversely, when the verb was printed in red, subjects had to refrain from moving (Fig. 2B). As in Experiment 1, we set the upper RT limit for go-trials to 600 ms. Overtime reaching-trials were kept for the analyses if they were shorter than 800 ms (less than 0.3% of the overall overtime reaching-trials were excluded). Each verb (12 verbs overall) was presented six times at each SOA, once in green and once in red. The experiment thus consisted of 288 trials, run in two blocks. Again, participants performed the task with the right arm.

#### 1.4.3. Experiment 3 (lexical discrimination task)

The procedure was identical to that described for Experiment 1, except that participants were required to move when a real word was presented and to refrain from moving when pseudo-words were shown (Fig. 2A). Each verb (48 overall: 24 real verbs and 24 corresponding pseudo-words) was presented five times for each SOA. The experiment thus consisted of 480 trials, run in four blocks. Participants always employed the right arm.

#### 1.4.4. Data analyses

For each participant, the mean RTs of correct trials and the mean percentages of errors were calculated for each verb category. The RT was determined as the time difference between the time of occurrence of the go-signal and movement onset. All the RTs of correct trials were included. We analyzed only the errors made during the go trials. Within this framework, we defined errors as those instances in which participants remained still on the central stimulus. We did not consider: i) errors on no-go trials (because in the semantic task they were related just to verbs whose meaning did not clearly involve movement of the effectors); ii) early responses, i.e. instances in which participants touched the monitor before the appearance of the central stimulus or instances in which they moved the arm while holding the central stimulus (about 1.45% of the overall trials); iii) missed responses, i.e. instances in which participants did not touch the central target at the beginning of the trial or they never moved their finger from it (about 0.26% of the overall trials). Both missed and early responses are indices of the attention that a given participant pays to the task and, crucially, they did not differ across the three tasks.

Repeated-measures ANOVAs were performed to assess differences in RTs and error rates with respect to the verb category and the two SOAs in the three experiments. Mauchley's test evaluated the sphericity assumption and, where appropriate, correction of the degrees of freedom was made according to the Greenhouse–Geisser procedure. Bonferroni correction was applied to all *post hoc* tests (pairwise comparisons). In order to control for the sample size, for each statistic we computed the eta-squared (η^2^), a coefficient that estimate the so-called “effect size”. An effect size is a measure used for describing the degree of relationship between dependent and independent variables independently of the sample size. Values of η^2^ higher than 0.14 indicate strong effect sizes, namely that the F-values obtained are unlikely to depend on the sample size. Values of η^2^ around 0.06 indicate medium effect size, and values smaller than 0.01 indicate small effect sizes [21]–[22].

In addition, a linear mixed model was employed to account for fixed and random effects [23]–[24]. This analysis allows exclusion of the possibility that any difference between verb categories could be due to variability embedded in the verbs chosen instead of being a genuine effect of verb category per se. We considered as fixed effects the factors verb category (mean RTs or mean percentage of mistakes obtained for neutral arm/hand, emotional arm/hand and neutral leg/foot verbs), and SOA (53.2 ms/332.5 ms) while the factors words (mean RT or mean percentage of mistakes obtained at each verb) and participants were considered random effects.

## Results

### 2.1. Semantic task

As a first step, we assessed the effect of verb categories on reaching movements by comparing the length of RTs and the error rates with a two-way repeated-measures ANOVA [factors: verb category (emotional arm/hand-related, neutral arm/hand-related and leg/foot-related verbs); and SOA (short: 53.2 ms, long: 332.5 ms)].

**Figure 3:**
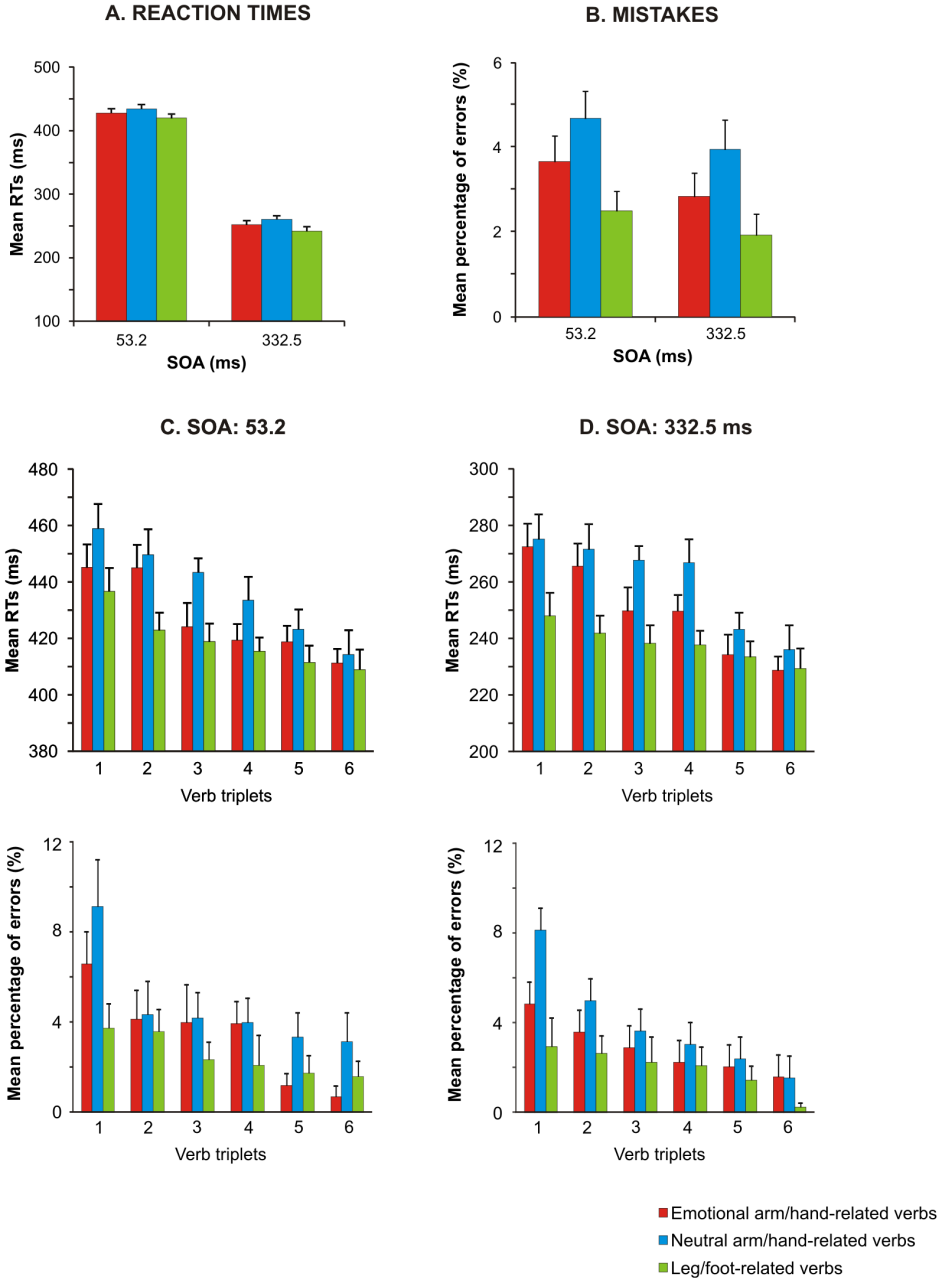
Effect of verb category on arm reaching movements in a semantics task. Average values of RTs (A) and mean percentage of errors (B) recorded when participants responded after reading neutral or negative valence arm/hand-related verbs, and neutral leg/foot-related verbs at a stimulus onset asynchrony (SOA) of 53.2 ms or 332.5 ms (see text for details of statistical analysis). Ranking of the mean RTs for each neutral or negative valence arm/hand-related and foot-related verb at a stimulus onset asynchrony (SOA) of 53.2 ms (C) and at an SOA of 332.5 m at each SOA (D).

For RTs (see Fig. 3A and Table 3) we found a main effect of verb category [F(2,58) = 43.6, p<0.0001, η2 = 0.11] and of SOA [F(1,29) = 1441.44, p<0.0001, η2 = 0.93]. In agreement with our previous results [7], we found that RTs to both categories of arm/hand-related verbs were significantly longer than those in response to leg/foot-related ones (pairwise comparisons, all p<0.001). This finding indicates once more the occurrence of an interference effect when participants perform a movement after reading a verb which describes an action involving the same effector used to give the response. However, in addition we also found that participants reacted significantly faster to negative valence arm/hand-related verbs than to neutral ones (pairwise comparison, p<0.05). The effect on the factor SOA was determined by a significant slowing of RTs when the go-signal was delivered at 332.5 ms after verb presentation (see Table 3).

**Table 3:** Summary of semantics task results.

		VERB CATEGORY		
SOA		Emotional arm/hand-related	Neutral arm/hand-related	Leg/foot-related
**53.2 ms**	RTs (ms)	427.84±6.4	434.3±6.5	419.6±6.1
	% Errors	3.66±0.6	4.69±0.65	2.5±0.47
**332.5 ms**	RTs (ms)	252.1±6.2	260.5±5.6	241.8±6.5
	% Errors	2.85±0.55	3.95±0.7	1.93±0.5

Mean (SEM) values of reaction times (RTs) and of rate of errors (% Errors) are reported for each verb category.

The analysis of the error rates (see Fig. 3B and Table 3) showed a main effect of verb category [F(2, 58) = 9.62, p<0.0001, η^2^ = 0.1]. As previously found [7], participants made more errors when they moved after reading arm/hand-related verbs (independently of their valence) than after reading leg/foot-related verbs (pairwise comparisons, all p<0.01). No significant differences emerged between emotional and neutral arm/hand-related verbs (pairwise comparisons, p = 0.21) even though the number of errors was smaller for the former than for the latter. Neither the SOA [F(1,29) = 3.2, p = 0.08, η2 = 0.02] nor the interaction between the two factors [F(2,58) = 0.04, p = 0.95, η2 = 0.001] reached significance.

In summary, the semantic task showed the emergence of an interference effect when participants executed reaching arm movements after the presentation of arm/hand-related verbs. However, on top of this we also found that the emotional connotation of verbs elicited a further modulation of the interference, given that reaching movements were executed faster after reading negative than neutral valence arm/hand-related verbs, and participants tended to make fewer errors in the former than in the latter case.

Importantly, the upper panels of figure 3C and 3D show that, as far as the overall RTs are concerned, both the modulations linked to verb categories were present at each item and at both SOAs, suggesting that this phenomena could not be due to the attributes of the chosen verbs. To assess statistically this finding, we analyzed the RTs using a linear mixed model considering both participants and verbs as random factors, while verb category and SOA were the fixed factors. We found that both fixed factors, but not their interaction, were significant [verb category: F(2,15) = 3.81, p<0.05; SOA: F(1,1030) = 9294.7, p<0.0001].

As error trials were repeated until a fixed number of correct responses was obtained, we performed the same item-by-item analysis done on mean RTs in order to investigate whether the average error percentage might reflect the same error performed again and again on the same verb. This was not the case, as shown in the bottom panels of Fig. 3C and 3D, because the mean percentage of errors was similar across the three verb categories even considering single items. The linear mixed-model analysis on the error rate (fixed factors: verb category, SOA and arm; random factors: participants and verbs) showed that only the factor verb category was significant [F(2,15) = 5.87, p<0.05].

All in all, these findings indicate that the differences we observed between the mean RTs or the mean percentage of errors among the three verb categories could not be ascribed to the variability embedded either in the words or in the participants selected.

### 2.2. Color discrimination task

In order to assess whether any sort of interference effect would be wiped out when the cue for solving the task was a non-linguistic one, we asked participants to respond or to withhold their movements according to the color in which verbs were printed.

**Figure 4:**
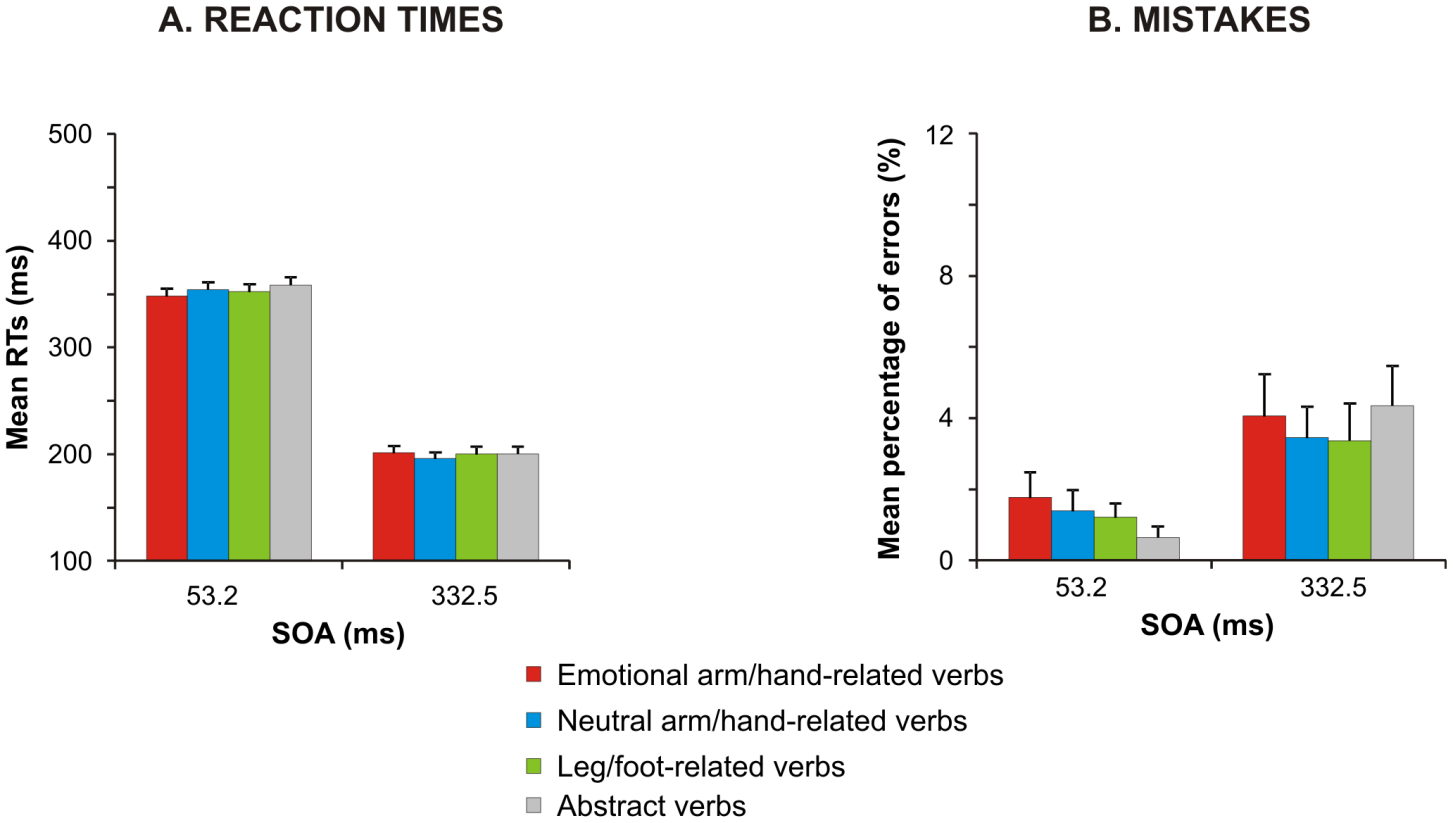
Effect of emotional meaning on arm reaching movements in the color discrimination task. Average values of RTs (A) and mean percentage of errors (B) recorded when participants responded after reading neutral or negative valence arm/hand-related verbs, neutral leg/foot-related verbs and no-effector-related verbs at a stimulus onset asynchrony (SOA) of 53.2 ms or 332.5 ms. See text for details of statistical analysis.

Exploiting the same two-way repeated-measures ANOVA design used before, we found that under this experimental condition neither RTs nor the percentage of errors changed according to the verb category (see Fig. 4 and Table 4).

**Table 4:** Summary of color discrimination task results.

		VERB CATEGORY			
SOA		Emotional arm/hand-related	Neutral arm/hand-related	Leg/foot-related	No-effector-related
**53.2 ms**	RTs (ms)	348±6.9	354.5±6.6	352.2±7.2	358.4±7.2
	% Errors	1.76±0.7	1.39±0.6	1.21±0.4	0.64±0.3
**332.5 ms**	RTs (ms)	201.1±6.3	195.9±5.9	200±7.2	200.4±6.7
	% Errors	4.1±1.2	3.4±0.9	3.37±1	4.36±1.1

Mean (SEM) values of reaction times (RTs) and of rate of errors (% Errors) are reported for each verb category.

In fact, the ANOVA on RTs showed no main effect of verb category [F(3,87) = 1.14, p = 0.3, η^2^ = 0.01] and no interaction [F(3,87) = 2.48, p = 0.07, η^2^ = 0.005]. This analysis revealed only a main effect of SOA [F(1,29) = 970.89, p<0.0001, η^2^ = 0.83], as RTs were slower when the go signal was given after the short than the long SOA (353.3±7 ms vs. 199.4±6.52 ms). The analysis of the error rate revealed no effects of verb category [F(3, 87) = 0.4, p = 0.76, η^2^ = 0.005] and no interaction [F(3,87) = 0.6, p = 0.6, η^2^ = 0.01], but there was a main effect of SOA [F(1,29) = 10.74, p<0.005, η^2^ = 0.13] due to a lower number of mistakes at the short than at the long SOA. Probably this was due to a trade-off between the speed of the response and its accuracy. Thus, when a non-linguistic feature was the cue for moving, all differences between verb categories disappeared.

### 2.3 Lexical discrimination task

Given that Sato et al. [8] showed that when a lexical task was administered to participants the interference effect disappeared, we aimed to replicate this result using a different list of verbs. In addition, we also wanted to assess whether and possibly how the negative connotation of verbs affects the RTs and the error rates of reaching movements under these experimental conditions.

**Figure 5:**
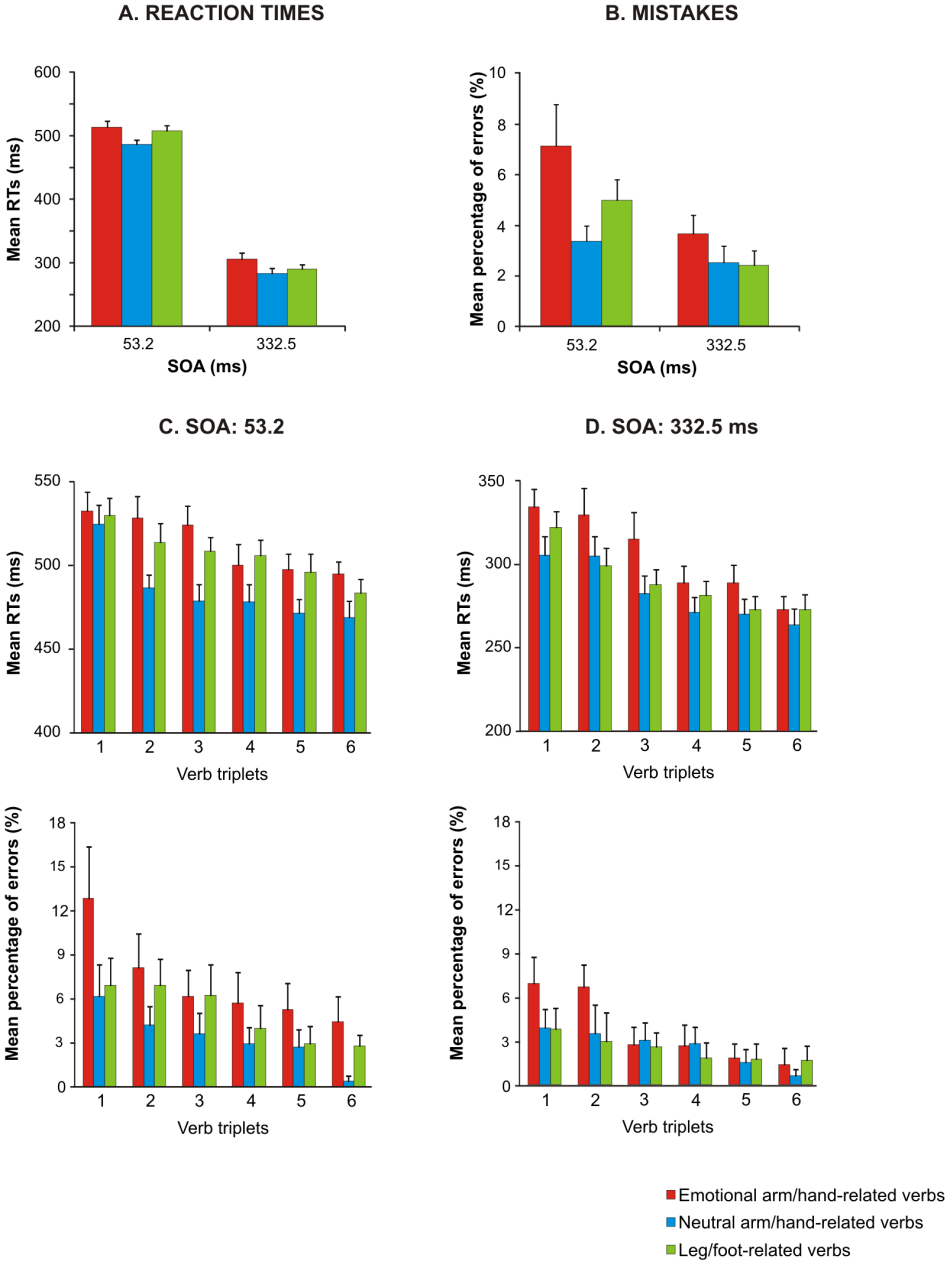
Effect of emotional meaning on arm-reaching movements in the lexical discrimination task. Average values of RTs (A) and mean percentage of errors (B) recorded when participants responded after reading neutral or negative valence arm/hand-related verbs and neutral leg/foot-related verbs at a stimulus onset asynchrony (SOA) of 53.2 ms or 332.5 ms. See text for details of statistical analysis. Ranking of the mean RTs for each neutral or negative valence arm/hand-related and foot-related verb at a stimulus onset asynchrony (SOA) of 53.2 ms (C) and at an SOA of 332.5 m at each SOA (D).

As shown in Figure 5A and Table 5, RTs were longer and the error rates were higher for emotional than for neutral arm/hand-related verbs and for leg/foot-related verbs.

**Table 5:** Summary of lexical task results.

		VERB CATEGORY		
SOA		Emotional arm/hand-related	Neutral arm/hand-related	Leg/foot-related
**53.2 ms**	RTs (ms)	513.1±9.5	484.9±8.2	506.6±8.2
	% Errors	7.11±1.6	3.35±0.6	4.97±0.8
**332.5 ms**	RTs (ms)	304.9±10.2	283±8.48	289.4±6.7
	% Errors	3.62±0.74	2.49±0.66	2.38±0.6

Mean (SEM) values of reaction times (RTs) and of rate of errors (% Errors) are reported for each verb category.

We compared the length of RTs and the error rates with a two-way repeated-measures ANOVA [factors: verb category (emotional arm/hand-related, neutral arm/hand-related and leg/foot-related verbs) and SOA (short: 53.2 ms, long: 332.5 ms)]. We found a main effect of the factor verb category [F(2,44) = 18.6, p<0.0001, η^2^ = 0.09] and of the factor SOA [F(1,22) = 1978.5, p<0.0001, η^2^ = 0.9]. Post hoc analysis (pairwise comparisons) revealed that participants were significantly slower after reading an emotional arm/hand-related verb than after either a neutral arm/hand-related or a leg/foot-related verb (p<0.05). In addition, participants showed significantly longer RTs after reading foot-related verbs than neutral hand-related verbs (p<0.005). The main effect of the factor SOA was due to the fact that overall participants were slower for the short than for the long SOA.

The error rate analysis (see Fig. 5B, Table 5) showed a main effect of verb category [F(2, 44) = 13.4, p<0.001, η^2^ = 0.11] and of SOA [F(1,22) = 4.33, p<0.05, η^2^ = 0.07], but not a significant interaction [F(2,44) = 2.6, p = 0.08, η^2^ = 0.03]. Participants made more errors after reading verbs with emotional meaning than after reading neutral arm/hand-related verbs (pairwise comparisons p<0.05). In addition, participants made more errors after the short than after the long SOA (p<0.005).

As for the semantic task, in order to exclude that the differences we found could be due to variability embedded in the chosen verbs, we performed the item-by-item analysis on both the mean RTs and the mean percentage of errors (fig. 5C and 5D). Again, we exploited a linear mixed model considering participants and verbs as random factors, and verb category and SOA as fixed factors.

As far as the mean RTs is concerned both fixed factor were significant [verb category: F(2,15) = 3.97, p<0.05; SOA: F(1,785) = 4352.1, p<0.0001]. Differently the analysis of the mean percentage of errors revealed that only the fixed factor SOA was significant [F(1,785) = 16.293, p<0.0001] while the factor verb category was not significant [F(2,15) = 316, p = 0.07]. However given that the pattern of the modulations linked to verb categories were very similar across single items (see fig. 5C and 5D bottom panels) and that the p value was very close to be significant, we believe, as for mean RTs, that the differences we observed between the mean percentage of errors should be truly related to the category of verbs.

Overall, the lexical task revealed a different type of interference based on the emotional valence of the verbs while for verbs with neutral valence revealed the existence of a facilitatory effect.

### 2.4. Comparisons across tasks

Since the designs of the three experiments were similar, we tried to provide a direct demonstration that indeed the effect of verb categories on reaching movements were fundamentally different as task demands change. To this end, we performed two separate two-way repeated-measures ANOVAs [factors: verb category (emotional arm/hand-related, neutral arm/hand-related and leg/foot-related verbs); and task (semantic task, color discrimination task and lexical task)] one on the RTs and the other on the error rates. Since previous analysis did not show interaction between SOA and verb category in any of the experiments, we collapsed over SOA both the RTs and the error rates. These analyses were performed only on the 23 participants that took part to all the experiments (Table 6).

**Table 6:** Summary of results obtained in each task after collapsing data over SOAs.

		VERB CATEGORY		
TASK		Emotional arm/hand-related	Neutral arm/hand-related	Leg/foot-related
**Semantic task**	RTs (ms)	334.4±4.9	344.6±4.3	324.6±5.4
	% Errors	3±0.5	4.6±0.8	2.2±0.5
**Color discrimination task**	RTs (ms)	268.7±6.7	269.2±5.6	268.3±6.3
	% Errors	2.8±0.9	2.2±05	2.2±0.6
**Lexical discrimination task**	RTs (ms)	409±8.9	383.9±7.8	398±6.9
	% Errors	5.4±1.1	2.9±0.5	3.7±0.6

Mean (SEM) values of reaction times (RTs) and of rate of errors (% Errors) are reported for each verb category.

The RT analysis showed a main effect of the factor task [F(1.53, 33.64) = 225.25, p<0.0001, η^2^ = 0.76] and of the factor verb category [F(2,44) = 6.65, p<0.005, η^2^ = 0.01]. Post hoc analysis (pairwise comparisons) on the factor task revealed that participants were significantly slowest when performing the lexical task and were fastest during the color discrimination task (all p<0.0001). The main effect of verb category was due to the fact that overall participants were significantly slower after reading an emotional arm/hand-related verb than after reading a leg/foot-related verb (pairwise comparisons, p<0.01). Crucially this analysis revealed a significant interaction between task and verb category [F(2,44) = 21.15, p<0.0001, η^2^ = 0.07]. Post hoc analysis revealed that all verb categories were significantly different in the lexical task and in the semantic task, but not in the color discrimination task. In the semantic task participants were slower after reading arm/hand-related verbs than a leg/foot-related verb (all p<0.005). However they exhibited shorter RTs after reading an emotional rather than a neutral arm/hand-related verb (p<0.005).

The error rate analysis (see Table 6) did not reveal any main effects [task: F(2, 44) = 1.95, p = 0.15, η^2^ = 0.06; verb category: F(2,44) = 2.875, p = 0.05, η^2^ = 0.01], but it showed a significant interaction [F(2,44) = 4.74, p<0.005, η^2^ = 0.1]. This interaction was due to i) the higher percentage of mistakes made by participants in the semantic task after reading verbs with neutral meaning than after reading emotional arm/hand-related verbs (pairwise comparisons p<0.05) or leg/foot-related verbs (p<0.005); and ii) the higher percentage of mistakes made by participants in the lexical task after reading verbs with emotional meaning than after reading neutral arm/hand-related verbs (pairwise comparisons p<0.05).

All in all these results provide a strong support for the existence of the different patterns observed in the analyses conducted on each task separately.

### 2.5. Movement times were never modulated

Reaching movements allow the measurement of movement times (MTs), which were determined as the time difference between time of movement onset and the time at which participants touched the peripheral target. This variable could potentially provide more information on the interaction between action-verb and motor cortex, so we analyzed the MTs as we did the RTs. However, as previously found [7], MTs were not modulated in any task (not shown). This could be either due to the fact that neural processes occurring during the execution of reaching movements are different from those occurring during their preparation or because of the greater variability of MTs than of RTs. It is very unlikely that the absence of the modulation of MTs could be due either to the relatively small distance of the target or because of the repetitive nature of the movement because in other experiments, in which peripheral stimuli were presented in the same position as the present one, we showed that MTs could be modulated according to the experimental context [25]–[26].

## Discussion

### Evidence for an interplay between motor responses and action language with neutral or negative connotation

This study shows for the first time that arm/hand-related verbs describing concrete actions with negative connotation modulate reaching movements in a different way from arm/hand- or leg/foot-related verbs describing actions with neutral connotation, provided that participants need to retrieve the semantics of the verb to solve the task. In fact, on the one hand, in the semantic task we found that reaching movements were slower and participants made more errors when they had to move after reading arm/hand-related verbs than after reading leg/foot-related verbs. These results nicely replicate our previous findings using different lists of verbs [7]. Crucially, on the other hand, we also found that the emotional connotation of arm/hand-related verbs further modulates the interference effect, speeding up RTs of reaching movements and decreasing the number of errors compared to neutral arm/hand-related verbs.

Firstly, these findings further support the link between action-related language and motor acts, suggesting that the motor system might play a role in understanding the meaning of an action word. By stating an involvement of the motor system, we do not want to exclude a priori that other brain regions participate in the processing of action-related word semantics. In fact, it is more likely that semantics could be processed by a distributed network of cortical areas encompassing both non-motor and motor regions (see [27]). As such, these data are compatible, at least, with the ‘weak’ versions of the embodied theory of language, which proposes that semantic representations are partly constituted by sensorimotor information [6].

It is noteworthy that it was recently claimed that go/no-go paradigms would not be suitable for testing the involvement of the motor system in action-language understanding (see [28]). In a go/no-go task in which the cues to move are words, first of all participants must recognize words, then retrieve their meaning and finally interpret the meaning according to the task rule in order to generate the appropriate response. Postle et al. [28] argued that, in this context, the high relatedness proportion of the go-condition (in our case 75%) would “induce expectancy sets that participants use to strategically enhance their performance in semantics matching tasks”. Therefore any influence on go-responses would be post-lexical and utilized solely to perform the required task. However, in our task we required participants to simply discriminate between concrete action-related and no-effector-related verbs. We never mentioned the presence of different categories of concrete verbs, nor did participants ever report having noticed this feature of the task. Furthermore, reaching movements were always the same, despite verb category. Thus, arguably, the expectancy or the level of alertness for ‘go’ conditions were surely higher than for the no-go conditions, but did not differ across different verbs categories. What influenced participants' performance was the relationship between the action described by the verb and the effector used to give the response. We agree that *per se* our results do not indubitably lead to the conclusion that the activity of cortical motor regions is causally related to action-language understanding. However, together with similar evidence from other behavioral studies (e.g. see [29]), from studies showing that processing of action-verb information depends on the integrity of the motor system [30]–[31] and from studies showing that somatotopic magnetic stimulation of the motor system specifically influence the processing either of action words [32] or of sentences describing actions [33], our findings support the notion that the motor system plays a role in understanding the meaning of an action word, possibly interacting with other areas.

Secondly, for the first time, we showed that the emotional valence of motor verbs affects the execution of actions, in a way that differs from to that driven by neutral motor verbs. In other words, the reenactment of motor schemas associated with the verb is likely to be affected by its valence. This effect strongly suggests the existence of a link between the processing of the emotional connotation of a verb and the processing of the motor information carried by its meaning. Importantly, in the semantics task, participants were explicitly required to discriminate only between action-related and no-effector-related verbs, i.e. they were not required to pay attention to the emotional valence of the verb. As a consequence, the emotional meaning was probably automatically integrated into the semantics analysis. Thus, it might be plausible that when participants are required to retrieve the semantics of the verbs, the motor system and the neural substrates involved in experiencing and understanding emotions (e.g. the limbic cortex, the amygdala) act in concert. Further studies implying recording of neural signals from the motor cortex during the execution of such a task might reveal whether the semantic processing of verbs with positive or negative valence increases or decreases the amount of motor activity with respect to the semantic processing of neutral verbs.

With respect to previous studies showing that the emotional connotation of words affects the execution of movements (e.g. [13], [34]–[35]) and the production of facial expression (e.g. [12]), our study provides more direct evidence in support of the embodiment of the emotional valence of words. Indeed, we compared the effect of verbs that had the same sensorimotor grounding but a different emotional valence. In other words, according to the embodied theory of language, the understanding of the verb's meaning required in both cases the activation of the corresponding motor schemas but, in the case of negative verbs only, the emotional experience evoked by the verb also had to be reenacted. In fact, our results indicate that when sensorimotor and emotional information coexists in the same verb, the understanding of its semantics relays on the reenactment of both the sensorimotor experience and of the emotional experience associated with the given action word.

It is noteworthy that the effects of verb categories on the speed and on the accuracy of reaching movement execution were the same at both SOAs. Thus, both the interference effect and the effect due to the emotional connotation of words occurred when the delay between the verb presentation and the go-signal was as little as 53.2 ms and did not end even when the SOA was 332.5 ms. These results are in full agreement with those of a previous study [7], and seem to indicate that verb processing could take place quickly, before the time thought to be sufficient to recruit frontal areas during reading [36] or word recognition [37]. An intriguing and testable possibility is that such a quick processing might be due to the automatic activation of the sensorimotor experience associated with the action described by the verb displayed. This is compatible with previous evidence showing that subliminally presented words automatically pre-activate essential parts of the cerebral networks recruited by language processing [38]–[39].

Finally, it has to be remarked that the overall amplitude of the interference effect is relatively small, in terms of both RTs and error rates. As we have pointed out in a previous work [7], this phenomenon represents a cost which is intrinsic to the way in which the neural network subserving action-language processing is built. To allow quick reactions in the presence of action-language material, this cost must not be too high. As a consequence, we expected that the modulation of the interference effect by the emotional valence of the verbs should have been even smaller, as it was. Nevertheless it was significant and very consistent across participants.

### When the cue for solving the task was the color of the printed verbs there was no effect on arm-reaching movements

To assess whether the interference between actions and verbs occurs only when the semantic content of a verb has to be retrieved, we ran two different control tasks. In the first one, the color discrimination task, participants had to move or withhold reaching movements according to a non-linguistic feature of verbs (i.e. the color of the printed verb). In agreement with our previous findings (see [7]), we did not find an interference effect, unequivocally indicating that the phenomenon takes place only when understanding of verbs' semantics is needed. In addition, under these experimental conditions, the emotional connotation of the verb did not affect the reaching movements at all. Similar results were obtained by Fruhholz et al. [40]. They instructed participants to name the color of neutral or negative verbs and they did not find any difference either in RTs or in the error rates between the two categories.

These findings indicate that valence of words affect actions only when their semantics have to be processed to solve the task. The same holds true as far as the interference effect is concerned. However, we do not exclude the possibility that even in this task participants read the verbs. It is likely, however, that in the color discrimination task the semantic analysis is not performed, being useless.

### The lexical discrimination task

We administered a lexical task as a further control of the results obtained in the semantic task because Sato et al. [8] showed that when employing a lexical task the interference effect disappeared.

In the lexical discrimination task, participants had to decide whether to move or to refrain from moving according to a linguistic rule (whether a word was sensible or not), which did not explicitly require understanding of the semantics of the verb. In fact, as Sato et al. [8] suggested, it is plausible that, as nonsense words differed from sensible verbs only by the replacement of one consonant in the first or second syllable, participants solved the task not by retrieving the semantics of the verbs but by processing their phonological features (see [41]). Indeed Yap et al [42] showed that lexical decisions may be influenced by many other variables, such as the number of contexts in which a word has been seen or the number of semantic neighbors. Thus, the extent of variability in the information associated with a word's meaning, i.e. its “semantic richness” [43], might influence in complex ways the outcome of the lexical task. However, it has been shown that while semantic classification's emphasis is on the word's meaning, lexical task classification's emphasis is on the word's form [42]. Consequently, the semantic and the lexical tasks have to be considered as different.

In agreement with the results of Sato et al. [8], we found that, in the lexical task, there were no differences in terms of either RTs or error rates between reaching movements executed after reading neutral arm/hand- and leg/foot-related verbs. However, we also found that RTs of reaching movements executed after reading negative valence arm/hand-related verbs were longer and their performance was less accurate than that shown after reading verbs of the other two categories. These results agree with those obtained by Kuperman et al [44], who found that negative valence of a word slows down lexical decisions with respect to words with neutral and positive valence, following a monotonic pattern.

We interpret this finding as follows. We employed verbs with negative emotional connotations and it has been shown that negative stimuli attract attention more efficiently than positive or neutral ones (e.g. see [15]–[16]). This is probably because the ability to detect information with negative emotional value and to respond with an appropriate action is critical for our survival, as it could prevent potential harm or unpleasant social interactions. In contrast, detection of positive stimuli (related to feeding or procreation) is less pressing [45]. This view is also known as the automatic vigilance hypothesis, and it holds that, according to the task at play, negative stimuli attract more attention (preferential engagement) or hold attention longer (delayed disengagement) than neutral or positive stimuli.

For instance, on the one hand it has been shown that when masked words are briefly presented, negative words are detected with better accuracy and higher sensitivity than positive or neutral ones [15], [46]. However, on the other hand it has also been shown that using an emotional version of the Stroop paradigm, naming the color of a neutral word is slower when it is preceded by a negative word than when preceded by a positive or neutral word [47]. These findings indicate that the effect of threatening stimuli on attention depends upon the task's rules. Consequently the effect of those stimuli on behavioral responses would also depend upon the experimental conditions. In fact, from an ethological point of view, threatening stimuli are very likely to exert different behavioral effects according to the situations. In some instances they might require quick actions, such as fleeing or fighting (e.g. when there is no other way to face a predator) but in other instances they might require freezing (e.g. when it is possible to hide from a predator).

In line with this hypothesis, Estes & Verges [35] showed that negative words elicit faster responses with respect to words with a positive meaning when participants were required to judge their valence, but when participants had to perform lexical decisions the same negative words slowed down responses. These results resemble the ones we obtained. In the semantic discrimination task the negative valence of the verbs probably attracted participants' attention, speeding up the semantic analysis and, consequently, improving response accuracy and shortening the RTs of arm-reaching movements. In the lexical discrimination task, the emotional connotation of the verbs probably still automatically attracted participants' attention but as it was not response-relevant, given that the task did not explicitly require semantics retrieval, it induced a slowing down of responses and an increase in the error rates.

In addition, we also found that in the lexical task RTs are shorter for leg/foot-related verbs than for neutral arm/hand-related verbs. These results overlap with those obtained by Sato et al [8], who also found that, in a lexical task, responses to neutral arm/hand-related verbs were faster than those to leg/foot action-related verbs by about 10 ms. However, this difference did not reach a statistical significance. Similarly, Scorolli & Borghi [48], using sentences that could refer either to an action usually performed with the hand (e.g. to throw the ball), or with the foot (e.g. to kick the ball), found a facilitation in responses given with the effector described in the language material. Thus one possible explanation of our data is that, only for verbs with neutral valence, a facilitatory effect occurs when the effector used is the same described by the verbs. However this effect is wiped away when the verb has a negative valence. Further experiments are needed to check this hypothesis.

Finally, it must be stressed that even though in both lexical and color discrimination tasks semantics analysis is not required, the lexical task still requires a structural analysis of the verb, whereas the color task requires just a perceptual analysis. In fact, the color discrimination task is far less difficult than the lexical task, as epitomized by the shorter RTs and fewer errors displayed by participants.

### Conclusions

All in all our results suggest that the comprehension of the emotional meaning of a verb implies the reenactment of the neural circuitry associated with the processing of the corresponding emotional experience as well as the reenactment of the corresponding motor schemas. In addition we showed that the processing of verbs' valence has a different impact on participants' motor performances. When the semantics of the verb must be retrieved, the automatic attraction of attention exerted by negative verbs allows faster processing of the semantic content and makes participants react faster and more accurately. When solving a lexical task, the automatic attraction towards emotional verbs impedes the decision-making process, increasing RTs and error rates, because the linguistic analysis required to solve the task requires not semantic but phonological analysis. Finally, when no linguistic analysis is required, as in the color discrimination task, no interference or modulation exerted by the emotional connotation occurs.

These differential task-related effects represent indirect evidence of the interplay between neural substrates processing emotions and motor responses during language processing (for evidence of such interplay outside the domain of language, see [49]). As such they are fully compatible with the embodied language hypothesis.

## References

[1] Fodor JA (1983) The modularity of mind. Cambridge, MA: MIT Press.

[2] Mahon BZ, Caramazza A (2005) The orchestration of the sensory-motor systems: Clues from Neuropsychology. Cogn Neuropsychol 22: 480–494. 21038262 10.1080/02643290442000446

[3] Barsalou LW (1999) Perceptual symbol systems. Behav Brain Sci 22: 577–660. 11301525 10.1017/s0140525x99002149

[4] Vigliocco G, Meteyard L, Andrews M, Kousta S (2009) Toward a theory of semantic representation. Lang Cogn 1: 219–248.

[5] Gallese V, Lakoff G (2005) The brain's concepts: the role of the sensory-motor system in conceptual knowledge. Cogn Neuropsychol 22: 455–479. 21038261 10.1080/02643290442000310

[6] Meteyard L, Cuadrado SR, Bahrami B, Vigliocco G (2012) Coming of age: a review of embodiment and the neuroscience of semantics. Cortex 48: 788–804. 21163473 10.1016/j.cortex.2010.11.002

[7] Mirabella G, Iaconelli S, Spadacenta S, Federico P, Gallese V (2012) Processing of hand-related verbs specifically affects the planning and execution of arm reaching movements. PLoS ONE 7: e35403 10.1371/journal.pone.0035403 22536380 PMC3335064

[8] Sato M, Mengarelli M, Riggio L, Gallese V, Buccino G (2008) Task related modulation of the motor system during language processing. Brain Lang 105: 83–90. 18054379 10.1016/j.bandl.2007.10.001

[9] Kousta ST, Vigliocco G, Vinson DP, Andrews M, Del Campo E (2011) The representation of abstract words: why emotion matters. J Exp Psychol Gen 140: 14–34. 21171803 10.1037/a0021446

[10] Vigliocco G, Kousta ST, Della Rosa PA, Vinson DP, Tettamanti M, et al (2013) The neural representation of abstract words: the role of emotion. Cereb Cortex 10.1093/cercor/bht025 23408565

[11] Newcombe PI, Campbell C, Siakaluk PD, Pexman PM (2012) Effects of emotional and sensorimotor knowledge in semantic processing of concrete and abstract nouns. Front Hum Neurosci 6: 275 10.3389/fnhum.2012.00275 23060778 PMC3465854

[12] Foroni F, Semin GR (2009) Language that puts you in touch with your bodily feelings: the multimodal responsiveness of affective expressions. Psychol Sci 20: 974–980. 19594858 10.1111/j.1467-9280.2009.02400.x

[13] Chen M, Bargh JA (1999) Consequences of automatic evaluation: immediate behavioral predispositions to approach or avoid the stimulus. Pers Soc Psychol Bull 25: 215–224.

[14] Havas DA, Glenberg AM, Rinck M (2007) Emotion simulation during language comprehension. Psychon Bull Rev 14: 436–441. 17874584 10.3758/bf03194085

[15] Dijksterhuis A, Aarts H (2003) On wildebeests and humans: the preferential detection of negative stimuli. Psychol Sci 14: 14–18. 12564748 10.1111/1467-9280.t01-1-01412

[16] Smith NK, Cacioppo JT, Larsen JT, Chartrand TL (2003) May I have your attention, please: electrocortical responses to positive and negative stimuli. Neuropsychologia 41: 171–183. 12459215 10.1016/s0028-3932(02)00147-1

[17] Oldfield RC (1971) The assessment and analysis of handedness: the Edinburgh inventory. Neuropsychologia 9: 97–113. 5146491 10.1016/0028-3932(71)90067-4

[18] Vigliocco G, Vinson DP, Druks J, Barber H, Cappa SF (2011) Nouns and verbs in the brain: a review of behavioural, electrophysiological, neuropsychological and imaging studies. Neurosci Biobehav Rev 35: 407–426. 20451552 10.1016/j.neubiorev.2010.04.007

[19] Bertinetto PM, Burani C, Laudanna A, Marconi L, Ratti D, et al. (2005) Corpus e Lessico di Frequenza dell'Italiano Scritto (CoLFIS).

[20] Bradley MM, Lang PJ (1999) Affective norms for English words (ANEW): instruction manual and affective ratings, Technical report C-1. Gainesville, FL: University of Florida.

[21] Cohen J (1988) Statistical power analysis for the behavioral sciences (2nd ed.). Hillsdale, NJ: Erlbaum.

[22] Lakens D (2013) Calculating and reporting effect sizes to facilitate cumulative science: a practical primer for t-tests and ANOVAs. Front Psychol 4: 863 10.3389/fpsyg.2013.00863 24324449 PMC3840331

[23] Clark HH (1973) The language-as-fixed effect fallacy: A critique of language statistics in psychological research. Journal of Verbal Learning and Behavior 12: 335–359.

[24] Raaijmakers JG (2003) A further look at the “language-as-fixed-effect fallacy”. Can J Exp Psychol 57: 141–151. 14596473 10.1037/h0087421

[25] Mirabella G, Pani P, Ferraina S (2008) Context influences on the preparation and execution of reaching movements. Cogn Neuropsychol 25: 996–1010. 19378414 10.1080/02643290802003216

[26] Mirabella G, Iaconelli S, Modugno N, Giannini G, Lena F, et al (2013) Stimulation of subthalamic nuclei restores a near normal planning strategy in Parkinson's patients. PLoS One 8: e62793 10.1371/journal.pone.0062793 23658775 PMC3643906

[27] Pulvermüller F (2013) Semantic embodiment, disembodiment or misembodiment? In search of meaning in modules and neuron circuits. Brain Lang 127: 86–103. 23932167 10.1016/j.bandl.2013.05.015

[28] Postle N, Ashton R, McFarland K, de Zubicaray GI (2013) No specific role for the manual motor system in processing the meanings of words related to the hand. Front Hum Neurosci 7: 11 10.3389/fnhum.2013.00011 23378833 PMC3561662

[29] Glenberg AM, Sato M, Cattaneo L, Riggio L, Palumbo D, Buccino G (2008) Processing abstract language modulates motor system activity. Q J Exp Psychol (Hove) 61: 905–919. 18470821 10.1080/17470210701625550

[30] Neininger B, Pulvemüller F (2003) Word-category specific deficits after lesions in the right hemisphere. Neuropsychologia 41: 53–70. 12427565 10.1016/s0028-3932(02)00126-4

[31] Boulenger V, Mechtouff L, Thobois S, Broussolle E, Jeannerod M, et al (2008) Word processing in Parkinson's disease is impaired for action verbs but not for concrete nouns. Neuropsychologia 46: 743–756. 18037143 10.1016/j.neuropsychologia.2007.10.007

[32] Pulvermuller F, Hauk O, Nikulin VV, Ilmoniemi RJ (2005) Functional links between motor and language systems. Eur J Neurosci 21: 793–797. 15733097 10.1111/j.1460-9568.2005.03900.x

[33] Buccino G, Riggio L, Melli G, Binkofski F, Gallese V, et al (2005) Listening to action-related sentences modulates the activity of the motor system: a combined TMS and behavioral study. Brain Res Cogn Brain Res 24: 355–363. 16099349 10.1016/j.cogbrainres.2005.02.020

[34] Solarz AK (1960) Latency of instrumental responses as a function of compatibility with the meaning of eliciting verbal signs. J Exp Psychol 59: 239–45. 13832584 10.1037/h0047274

[35] Estes Z, Verges M (2008) Freeze or flee? Negative stimuli elicit selective responding. Cognition 108: 557–565. 18433742 10.1016/j.cognition.2008.03.003

[36] Hauk O, Pulvermuller F (2004) Neurophysiological distinction of action words in the fronto-central cortex. Hum Brain Mapp 21: 191–201. 14755838 10.1002/hbm.10157 PMC6872027

[37] Hauk O, Davis MH, Ford M, Pulvermuller F, Marslen-Wilson WD (2006) The time course of visual word recognition as revealed by linear regression analysis of ERP data. Neuroimage 30: 1383–1400. 16460964 10.1016/j.neuroimage.2005.11.048

[38] Dehaene S, Naccache L, Cohen L, Bihan DL, Mangin JF, et al (2001) Cerebral mechanisms of word masking and unconscious repetition priming. Nat Neurosci 4: 752–758. 11426233 10.1038/89551

[39] Naccache L, Gaillard R, Adam C, Hasboun D, Clemenceau S, et al (2005) A direct intracranial record of emotions evoked by subliminal words. Proc Natl Acad Sci U S A 102: 7713–7717. 15897465 10.1073/pnas.0500542102 PMC1140423

[40] Frühholz S, Jellinghaus A, Herrmann M (2011) Time course of implicit processing and explicit processing of emotional faces and emotional words. Biol Psychol 87: 265–274. 21440031 10.1016/j.biopsycho.2011.03.008

[41] Fadiga L, Craighero L, Buccino G, Rizzolatti G (2002) Speech listening specifically modulates the excitability of tongue muscles: a TMS study. Eur J Neurosci 15: 399–402. 11849307 10.1046/j.0953-816x.2001.01874.x

[42] Yap MJ, Tan SE, Pexman PM, Hargreaves IS (2011) Is more always better? Effects of semantic richness on lexical decision, speeded pronunciation, and semantic classification. Psychon Bull Rev. 18: 742–50. 21494916 10.3758/s13423-011-0092-y

[43] Pexman PM, Hargreaves IS, Siakaluk P, Bodner G, Pope J (2008) There are many ways to be rich: Effects of three measures of semantic richness on word recognition. Psychonomic Bulletin & Review 15: 161–167. 18605497 10.3758/pbr.15.1.161

[44] Kuperman V, Estes Z, Brysbaert M, Warriner AB (2014) Emotion and language: Valence and arousal affect word recognition. J Exp Psychol Gen 143: 1065–81. 24490848 10.1037/a0035669 PMC4038659

[45] Pratto F, John OP (1991) Automatic vigilance: the attention-grabbing power of negative social information. J Pers Soc Psychol 61: 380–91. 1941510 10.1037//0022-3514.61.3.380

[46] Nasrallah M, Carmel D, Lavie N (2009) Murder, she wrote: enhanced sensitivity to negative word valence. Emotion 9: 609–618. 19803583 10.1037/a0016305 PMC2759814

[47] McKenna FP, Sharma D (2004) Reversing the emotional Stroop effect reveals that it is not what it seems: the role of fast and slow components. J Exp Psychol Learn Mem Cogn 30: 382–392. 14979812 10.1037/0278-7393.30.2.382

[48] Scorolli C, Borghi AM (2007) Sentence comprehension and action: effector specific modulation of the motor system. Brain Res 1130: 119–24. 17174278 10.1016/j.brainres.2006.10.033

[49] Ferri F, Ebisch SJ, Costantini M, Salone A, Arciero G, et al (2013) Binding action and emotion in social understanding. PLoS ONE 8: e54091 10.1371/journal.pone.0054091 23349792 PMC3547946

