# Correction: Modulation of Arm Reaching Movements during Processing of Arm/Hand-Related Action Verbs with and without Emotional Connotation

**DOI:** 10.1371/journal.pone.0116563

**Published:** 2014-12-22

**Authors:** The PLOS ONE Staff

There are errors in Figure 2 and its legend. In the figure, it appears that subjects had to move when the verb was printed in red and to refrain from moving when it was printed in green; however, as described in the text (see paragraph 1.4.2), it is the opposite. In the Figure 2 legend, panel A should correspond to the "Lexical task" and panel B to the "Color discrimination task.” The authors have provided a corrected Figure 2 and legend here.

**Figure 2:**
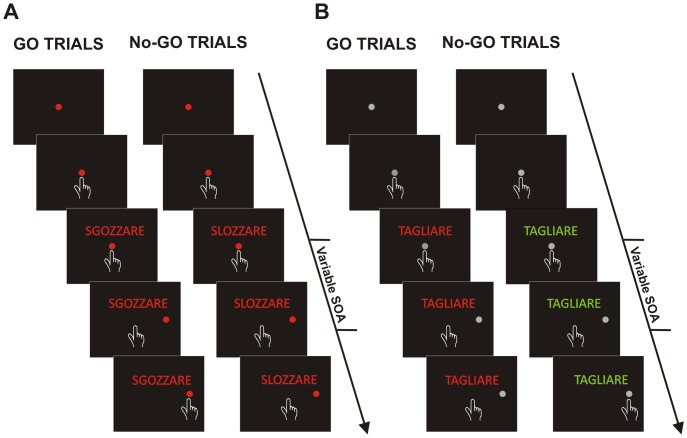
Schematic representation of control tasks. (A) Lexical task. Each trial started with the presentation of a red central target that participants had to touch and hold for a variable period. After a variable SOA a peripheral target appeared and participants were asked either to touch it if it was a real word (go-trials) or to stay still if it was a pseudo-word (no-go trials; see Methods for more details). **(B)** **Color discrimination task.** Each trial started with the presentation of a grey central target that participants had to touch and hold for a variable period. After a variable delay (stimulus onset asynchrony; SOA) a peripheral target appeared and participants were asked either to touch it, if it was printed in green (go-trials) or to stay still if it was printed in red (no-go trials; see Methods for more details).
